# Virtues in Competency-Based Assessment Frameworks: A Text Analysis

**DOI:** 10.5334/pme.996

**Published:** 2023-10-19

**Authors:** Pleuntje M. B. Verstegen, J. J. (Jos) Kole, A. Stef Groenewoud, Frank J. A. van den Hoogen

**Affiliations:** 1 Radboud university medical center, Radboud Institute for Health Sciences, Nijmegen, The Netherlands

## Abstract

**Introduction:**

Official documentation of specialty training provides comprehensive and elaborate criteria to assess residents. These criteria are commonly described in terms of competency roles and entrustable professional activities (EPA’s), but they may also *implicitly* encompass virtues. Virtues are desirable personal qualities that enable a person, in this case, a medical specialist, to make and act on the right decisions. We articulate these virtues and explore the resulting implied ideal of a medical professional.

**Method:**

We applied a two-staged *virtue ethical content analysis* to analyze documents, specific to the Dutch training program of the Ear, Nose, and Throat (ENT) specialty. First, we identified explicit references to virtues. Next, we articulated implicit virtues through interpretation. The results were categorized into cardinal, intellectual, moral, and professional virtues.

**Results:**

Thirty virtues were identified in the ENT- training program. Amongst them, practical wisdom, temperance, and commitment. Furthermore, integrity, curiosity, flexibility, attentiveness, trustworthiness and calmness are often implicitly assumed. Notable findings are the emphasis on efficiency and effectiveness. Together, these virtues depict an ideal of a future medical specialist.

**Conclusion:**

Our findings suggest that competency-frameworks and EPA’s implicitly appeal to virtues and articulate a specific ideal surgeon. Explicit attention for virtue development and discussion of the role and relevance of implied ideal professionals in terms of virtues could further improve specialty training.

## Introduction

How can we best train and assess a future medical specialist? The common reply can be found in the paradigm of competency-based medical education (CBME), as formulated in the Canadian Medical Education Directives for Specialists-framework (Can-MEDS) and the more recently developed entrustable professional activities (EPA’s) [1,2,3,4,5]. These two approaches provide comprehensive assessment criteria and milestones to evaluate and assess a resident’s performance, but they may also implicitly encompass virtues. To become a good professional implies more than becoming competent. It also implies that one *develops* positive personal qualities, virtues, that dispose you to make the right decisions and act accordingly [6]. This view of developing desirable personal qualities is central to the approach of virtue ethics. Within this approach, a person acquires virtues through a combination of habituation, role-modeling and reflection [7]. Virtues enable people to do the right thing, for the right reason, in the right way, and at the right moment [8].

The importance of virtue ethics for medicine is widely acknowledged [9,10] and its relevance for medical education and teaching professionalism is also recognized [11,12,13], such as in publications about ‘the good doctor’ [14,15,16]. However, the application of a virtue ethics’ analysis to educational documents is lacking although they often figure implicitly in such documents.

CBME has been thoroughly analysed and criticised in literature [17,18,19], and its particular discourse as well [20,21]. The differences between written CanMEDS roles and the construction of these roles in reality and the relation of these roles to professional identity have been studied in depth [22,23].

Yet, the question remains what such written requirements themselves imply and presuppose in terms of desired personal qualities, that is, virtues. To exemplify, the CanMEDS role of collaborator states that a resident should be able to “*implement strategies to promote understanding, manage differences, and resolve conflicts*” [2]. This description presupposes the personal qualities – virtues – of empathy and tactfulness.

Next to implying specific personal qualities, written training programs also include an ethical dimension since a good medical professional should adhere to professional and ethical standards. Yet, what is implicitly ethically required of residents in training programs (e.g. through the acquisition of certain virtues), seems to be rarely explicitly justified and is often stated in general and intuitive terms., e.g. *“[apply] best practices and [adhere] to high ethical standards*” [2]. Taken together, if required virtues and ethical standards remain implicit and hidden in text, they are unlikely to become the subject of discussion and evaluation. Furthermore, they run the risk of misinterpretation or of implying a possibly undesirable type of moral standard to which a specialist should adhere. This study therefore aims to answer the following questions: i) which virtues are, implicitly and explicitly, embedded in specialty training programs, and ii) what sort of good medical professional results from these set of virtues?

## Method

We conducted an extended version of qualitative content analysis to identify implicit and explicit virtue references in CBME documents and applied it to the revised national training program for Dutch Ear, Nose, and Throat, and Head and Neck surgery residents, ENTER2 [24].

### Method Selection and Extension

**Figure 1:**
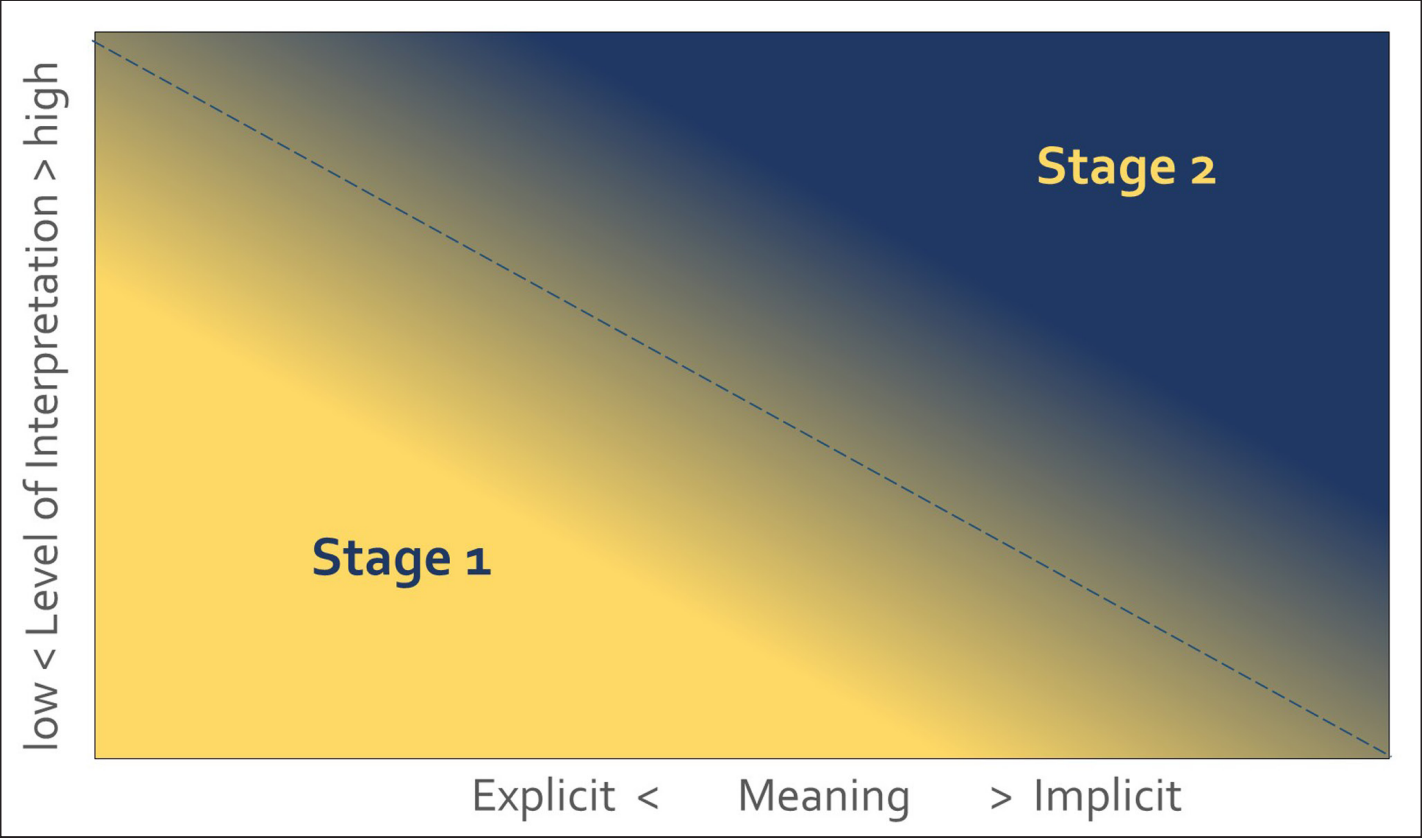
Schematic representation of Virtue Ethical Content Analysis.

To analyze CBME documents and identify implicit and explicit desiderata in terms of virtuous character traits, we felt it was necessary to refine currently available methods. Content analysis focuses on the analysis of the meaning of *text data* [25], but by itself, this method does not offer tools for identifying virtues. In addition, in the field of empirical virtue ethics, clear instructions or specific criteria to identify virtue expressions in text seem absent too [26,27,28]. Where criteria are given, they are limited to recognizing explicit virtue words [29]. Phrases and text passages may also refer indirectly to virtues. In such cases, guided interpretation is necessary. For that reason, we created two stages of analysis that correspond with the two levels of textual meaning, namely, explicit and implicit meaning (see Figure 1). Our applied method features an *extended* version of content analysis because our guided interpretation depends on virtue ethical theory. We call this refined method, *virtue ethical content analysis*.

### Case Selection

We analyzed the Dutch revised training program of the Ear, Nose, and Throat, and Head and Neck surgical specialty, ENTER2 [24]. The ENT specialty has ample experience with CBME [30]. ENTER2 features both novel and elaborate assessment criteria. This training program is, therefore, a representative example of contemporary CBME specialty training programs. It provides extensive and precise criteria to evaluate and assess residents’ performance.

**Figure 2:**
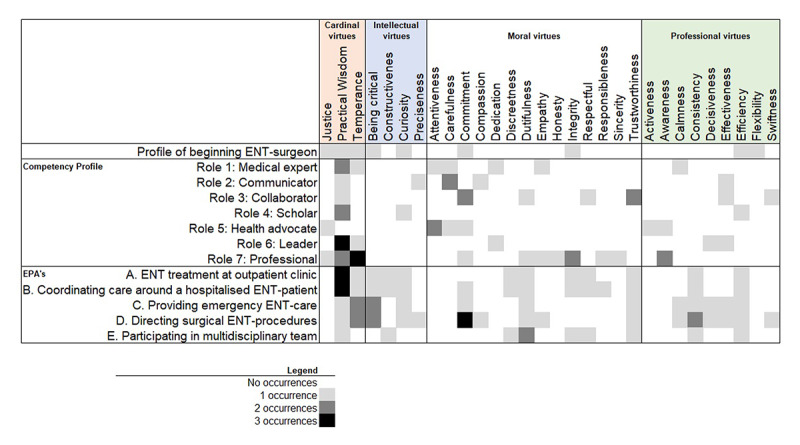
Explicit and implicit virtues of ENTER2.

We selected the ENTER2 chapters that describe the profile of a graduated ENT surgeon, the seven roles of the CanMEDS-profile (59 competences), and the attitude and behavior competences listed in the five EPA’s (61 criteria). See Figure 2 for an overview of selected sections and their titles.

### Coding and Analysis

We used Atlas.TI software for coding and analyzing the selected chapters of ENTER2. A paragraph of each selected section of ENTER2 was independently coded by a second researcher. The researchers studied each other’s codes and discussed these until a consensus was reached. Both researchers are philosophically trained and familiar with virtue ethics. During the iterative process of coding and analyzing, it appeared necessary to distinguish between explicit virtue references and implicit virtue references. Hence, we devised two stages of analysis. Still, under what conditions a text passage can be labelled as referring to virtue was lacking. We then further discussed and devised specific conditions for each stage to provide actual and understandable criteria to guide the analysis and stay faithful to the richness of virtue ethics and to avoid reductionism of its central concept. The concept of virtue closely relates to personal development, and goes beyond simple analyzable entities in texts. As a result, the conditions that we specify to identify textual references to virtue are numerous. We based them on writings of representative virtue ethics experts like Annas [8], MacIntyre [31], Marcum [9], Pellegrino [16] and Carr [13]. Ideally, other researchers than we should be able to identify similar test passages as references to virtues by applying the criteria.

The first researcher kept a log to record all methodological issues and decisions. These issues were discussed and resolved in team meetings. It should be noted that authors of training programs such as ENTER2 might have been unaware of implicit or presupposed virtue ethics. Our interpretation of the results of our analysis, therefore, does not represent the intentions of the authors of ENTER2. Nevertheless, our analysis raises awareness of the role and relevance of virtues for medical education and paves the way for reflection and discussion.

### Stage 1: Explicit Virtue References

In the *first stage*, we have identified terms in the text that are known to refer directly and literally to virtues. This stage parallels the directed approach of qualitative content analysis [25,32]. We derived three conditions from the previously mentioned virtue ethical literature to delineate which terms refer directly and literally to virtues:

1. The term should pass what we call ‘the commonsense question test’: “Can a person, as person, be praised by this qualification?”, because virtues are praiseworthy *personal* qualities.
2. The term should be mentioned in the context of an action or activity because a virtue is about *acting* in the right way [8].
3. The term resembles those virtues that figure in well-known but non-conclusive lists of virtues, that are mentioned in virtue ethics literature.

Fox example, in ENTER2 we read the word *adequate*. We did not code *adequate* as virtue because it fails to meet these conditions. In contrast, we included the word *attentive* as virtue since it meets all conditions in the given context. Note that there is neither a fixed number nor an absolute list of personal qualities that are considered virtues. Thus, this third condition is not a necessary condition: a word can still be identified as a virtue if it does not appear on well-known virtue-lists because these lists are non-exhaustive. Besides the previous stated literature, our list is based on enumerated virtues as found in Pellegrino and Thomasma [33], Kotzee, Ignatowicz [34], Snow and Oakley [35].

### Stage 2: Implicit Virtue References

Virtues may also be expressed in a less literal way in phrases and passages that refer indirectly to virtues. In such cases, we needed to interpret text data for their implied *meaning*. In the *second stage* of our analysis, we identified implied virtues through content analysis with a more hermeneutical approach. Hermeneutics, a philosophical approach, focuses on the articulation of meaning through interpretation [36,37]. Here again, we have inferred identification criteria from the aforementioned virtue ethics literature. Larger text parts, such sentences or passages, are considered to refer indirectly to virtues if they meet at least two out of seven conditions. To count as reference to a virtue, text passages should meet more than one out of these conditions, although not necessarily the same characteristics on each instance. The text part should refer to: (1) to a quality of a person, (2) that is desirable or positive, (3) and continuous in time (part of someone’s personality through time). (4) It should have an intentional stance or a sense of direction, (5) refer to intrinsic motivation or a longing to excel. (6) It should refer to action or an activity, and (7) relate to an emotion or feeling.

### Structuring Identified Virtues

After identifying specific virtues in the training program, we looked for themes to categorize them. It appeared helpful to use well known categories, already available in virtue ethical theory. We distinguish between cardinal, intellectual, and moral virtues. This classification dates back to Ancient Greek philosophers such as Plato and Aristotle [38]. Some of the virtues identified did not fit into any of these classic categories. We labelled them as professional virtues because these virtues resemble qualities deemed laudable in the profession and practice of the ENT surgeon. See Table 1 for more information about these virtue categories which are based on literature [9,38,39]. Although such categorization is also widely debated, these four categories served the purpose of providing an insightful overview. Furthermore, any categorization can lead to discussion, as it is almost never completely exhaustive.

**Table 1:** Categories of virtues.


CATEGORY	DESCRIPTION	SPECIFIC VIRTUES

**Cardinal Virtues**	The four main or most important virtues throughout history [40]. These virtues are presupposed in all other virtues [41].	Practical wisdom Courage Justice Temperance

**Intellectual Virtues**	Virtues that enable acquisition of knowledge and to understand truth *(sometimes also labelled as epistemic virtues)* [9,38,39].	e.g. Curiosity, Preciseness

**Moral Virtues**	Virtues that make people take concern for others, virtues distinctive of a moral person. *(sometimes also labelled as social virtues*) [9,38,39].	e.g. Empathy, Integrity

**Professional Virtues**	Virtues required to excel in a profession. In this case, character traits to be a good physician, other than those in other categories.	e.g. Efficiency, Flexibility


## Results

We identified a wide variety of explicitly (literal) and implicitly (less literal) present virtues. The authors of ENTER2 used 18 different virtue-like terms and implicitly referred to 12 other distinctive virtues. The frequency of identified virtues ranges from one to three occurrences in one competency role or EPA. An overview of the results is offered in Figure 2 (literal references to virtues are not always nouns, but for clarity reasons we present all identified virtues in the figure as nouns).

### Cardinal Virtues

Three out of four cardinal virtues, namely practical wisdom, temperance and justice, were identified in ENTER2 with practical wisdom most often referenced (21 times). This virtue refers to the ability to judge what is the right thing to do in each new specific situation and to act accordingly [10]. Practical wisdom can be found in various sections of the program, for instance when the competence of medical expertise is described as: *“Stays calm, acts in the right way and chooses his priorities correctly.”* [24, p. 43]. Practical wisdom is also often assumed in competences where resolute action is combined with proper judgement of what the situation demands. Additionally, the entire training program appears to appeal indirectly to the need of practical wisdom by the frequent use of words like ‘adequate’ and ‘accurate’, and less frequently, ‘right’, ‘good’, ‘fitting’ and ‘the right way’. These words presuppose normative standards of good and bad and the ability to judge wisely in accordance with them.

The cardinal virtue of temperance also often occurs throughout ENTER2 (12 times). Temperance requires self-knowledge and the ability to moderate one’s desires [9]. It is assumed whenever the resident must balance between excess and deficiency. Temperance is, for example, in play when a resident is responsible for care of a hospitalized patient (EPA B), and should “*limit[s] time and attention in task performance in proportion to workload, responsibilities, and urgency”* ([24, p. 49]. Temperance is also implicitly demanded during emergency care (EPA C) when residents are expected to control their emotions and remain focused.

Justice, the third cardinal virtue, is rarely mentioned. When ENTER2 refers to this virtue, justice seems to convey a dual meaning. In the case of demonstrating the role of health advocate (role 5), a resident should campaign for the accessibility of healthcare to everyone. Simultaneously, however, the resident should be aware of healthcare benefits and costs and divide these proportionally.

### Intellectual Virtues

Intellectual virtues enable the proper acquisition of knowledge [42]. The virtues of being critical, constructiveness, curiosity and preciseness are most often mentioned within the EPA’s. Being critical is mentioned, for example, when residents are expected to perform surgical procedures independently (EPA D), to initiate discussions to improve their own and others’ performance, and to adapt to or give feedback ([24, p. 53]. This description seems to imply a willingness to learn and to be critical of one’s own actions. Unexpectedly, intellectual virtues are rarely identified within the roles of medical expert or scholar.

### Moral Virtues

Moral virtues are the personal qualities that make specialists moral persons and ethical responsible professionals. The training program articulates, often only once, 14 different moral virtues. These include well-known character traits such as honesty, compassion, and sincerity [43]. A resident is also expected to ‘act with integrity’, which directly refers to the virtue of integrity. Residents are also often expected to commit themselves to agreements and protocols and to fulfil their duties. Each EPA formulation states, for example: *“Acts in accordance with the Medical Treatment Contracts Act”* ([24, p. 55].

Interestingly, the virtue of carefulness is most prominent in the description of the role of communicator. Being careful is frequently demanded while residents coordinate communication. They should pay attention to proper information exchange between themselves and other caretakers, patients, and their families and they are expected to be thoughtful of their “*needs, expectations, fears and hopes*” ([24, p. 43].

### Professional Virtues

The training program also introduces contemporary praiseworthy qualities that have no heritage in classical virtue ethical theory but are currently considered praiseworthy in the pursuit of the profession. It is repeatedly emphasized that a resident needs to be efficient, consistent, and efficacious (see Figure 2 for place and frequency). Competences often indicate the awareness of moderate use of means, acting consistently over time, and being focused on ends. For example, a resident should be *efficacious* in his or her CanMEDS-role as communicator when managing relations with the patient and their environment. However, it is never explicitly mentioned on which ends the resident should focus. Other professional virtues articulated in the text are calmness, decisiveness and swiftness (EPA C-D). Interestingly, the profile of a beginning ENT-surgeon describes the main objective of residency training as follows: “*A communicative competent, flexible person with integrity who is constantly observant towards improving one’s own and joint functioning*” [24, p. 6]. Flexibility, awareness, and activeness refer, of course, to virtues.

### Resulting Portrayal of a Good Medical Professional

Taken together, these virtues offer a character sketch of an ENT surgeon. In general, the training program indirectly requires residents to become practically wise, temperate, consistent and efficient, and to be committed and dutiful. Furthermore, they should also be trustworthy, curious, emphatic, careful, calm, and aware. They should also display integrity and be attentive towards others. Together, these virtues offer a portrayal of a good medical professional, specifically a good ENT-surgeon. This portrayal functions as an ideal.

## Discussion

Our refined method of virtue ethical content analysis enables explication of virtues in competency based assessment frameworks. We identified explicit and implicit virtue references in the Dutch national training program for future Ear, Nose, and Throat surgeons. With this study we aim to contribute to the reflection and discussion of what a good doctor entails and what CBME presupposes or implies of residents. Such reflection and discussion may support further development of postgraduate education. In this discussion, we will discuss the most interesting results per category from a total of 30 different virtues identified in ENTER2.

### The Ideal Virtuous Surgeon?

The category of *cardinal* (main) virtues is well-represented in the training program although the virtue of courage is never mentioned. This might seem unusual given that surgeons face difficult – even life threatening – situations that seem to demand courage. The high frequency of the cardinal virtue of practical wisdom accords well with medical-ethics literature. Numerous studies stress the importance of practical wisdom for medical practice, and it generally considered an essential virtue for physicians [44,45,46]. However, the refence to this virtue in ENTER2 is ambivalent. On the one hand, the emphasis on practical wisdom suggests that surgeons should judge for themselves what to do. It also suggests that the level of experience influences the quality of such judgement. On the other hand, the program often emphasizes adherence to procedures and rules that may conflict with one’s own professional judgement.

Both categories of *intellectual* and *moral* virtues feature well-known praiseworthy qualities such as attentiveness, trustworthiness and integrity. However, sometimes the appeal to certain virtues seems rather arbitrary. For instance, ENTER2 refers to the virtue of being critical in the EPA’s of providing emergency care or coordinating care while one might expect this virtue first and foremost in the context of the role of medical expert.

The virtues of honesty and compassion are rarely mentioned, suggesting that these virtues are less valued for ENT-surgeons. Yet, this would contrast with literature about ‘the virtuous physician’ that presents compassion as vital quality for good physicians [47,48].

*Professional* virtues do root in classic virtue ethical literature, but these virtues reflect characteristics of past and current medical practice. The professional virtues articulated in ENTER2 represent values that are currently highly valued in medical practice, such as efficacy, consistency and efficiency. The latter seems linked to the scarcity of resources like time and money. Although this is actual practice and predicament, training program developers and educators could discuss whether they want to stress such efficiency. Focus on this virtue may jeopardize other qualities that are important for good doctoring.

Furthermore, the authors repeatedly call on residents to make the *right* choices and take *good* action. Descriptions of competence frequently stress to improve overall performance. This continuous drive to improvement strongly resembles the focus of virtue ethics on excellence [8].

### Strengths and Limitations

The main strength of this study is its shift of attention from explicitly stated assessable qualifications to the implied virtues that residents should acquire as well. This study illustrates how a virtue ethical approach may fit the tone of residency training and how it may enrich reflection on personal and professional development during this training. Furthermore, this study contributes to empirical virtue ethics by providing specific criteria to guide analysis of explicit and implicit virtue references in texts. Our refined analysis method may be of value in other educational fields that use extensive profiles and assessment frameworks in order to establish someone as proverbially fit to fly.

This study also has several limitations. One limitation concerns the definition of the concept of virtue. This definition remains the topic of continuous theoretical debate. Consequently, our identification of virtues remains disputable as well. Another limitation is that this study restricts itself to the analysis of only one training program. A natural follow up would be to apply the method of virtue ethical content analysis to other training programs as well.

### Recommendations

We have several recommendations. Our analysis is a stepping stone to consciously reflect, discuss and evaluate the virtues that residents in different medical domains should acquire. The literature describes virtues for physicians [9,10,49], but other stakeholders such as educators and patients may also be involved in determining which virtues surgeons and other medical professionals should have. In addition, future research can address questions such as ‘are there specialty specific virtues?’ And, once all stakeholders have agreed on which virtues are important, how can they be cultivated in a conscious and effective way during workplace-based training?

To continue, ENTER2 prescribes training objectives, but gives little guidance on how to achieve them. If we take the indirect call for a virtuous surgeon seriously, we could adapt training programs to make them more focused on personal and professional development. One recommendation might be to offer stages of development, to establish an order that prioritizes certain virtues or competences and to discard possible conflicting requirements.

If the training program is adapted in this way, it will result in a more balanced program which is better aligned with lived workplace-based residency training.

## Conclusion

This study analyzes both explicitly as well as implicitly mentioned virtues in a current assessment framework for future ENT surgeons. The analysis of the explicit and implicit virtue ethical content shows that there is an implicit normative ideal of a good medical professional wrapped in competency language. Such findings pave the way for reflection and discussion of such ideals to improve the scope and message of CBME programs.

The current portrayed ENT surgeon must have acquired various cardinal, intellectual, moral and professional virtues. Next to competency-based medical specialty training, there is a unique opportunity to train professionals as a person in order to provide them with the acquisition of the right virtues to excel in their profession. Explicit attention to virtues may enrich medical specialty training.
